# Oxidative Biochemistry Disbalance and Changes on Proteomic Profile in Salivary Glands of Rats Induced by Chronic Exposure to Methylmercury

**DOI:** 10.1155/2017/5653291

**Published:** 2017-07-24

**Authors:** Leonardo Oliveira Bittencourt, Bruna Puty, Senda Charone, Walessa Alana Bragança Aragão, Paulo Mecenas Farias-Junior, Marcia Cristina Freitas Silva, Maria Elena Crespo-Lopez, Aline de Lima Leite, Marilia Afonso Rabelo Buzalaf, Rafael Rodrigues Lima

**Affiliations:** ^1^Laboratory of Functional and Structural Biology, Institute of Biological Sciences, Federal University of Pará, Belém, PA, Brazil; ^2^Department of Biological Sciences, Bauru Dental School, University of São Paulo, Bauru, São Paulo, SP, Brazil; ^3^Laboratory of Molecular Pharmacology, Institute of Biological Sciences, Federal University of Pará, Belém, PA, Brazil

## Abstract

Methylmercury (MeHg) is one of the most toxic mercury species, which can cause many systemic damages, but little is known about its effect in the salivary glands. This study aimed to analyze the mercury levels, oxidative stress, and proteomic profile in parotid, submandibular, and sublingual salivary glands of rats, after chronic MeHg intoxication. Two groups of twenty male Wistar rats (90 days of age) were used on the experiment. MeHg group was intoxicated by intragastric gavage with MeHg at a dose of 0.04 mg/kg/day for 60 days, while the control group received only oil. After the period of intoxication, the glands were collected for evaluation of total mercury levels, proteomic profile, and oxidative balance by analyzing the antioxidant capacity against peroxyl radicals (ACAP), lipid peroxidation (LPO), and nitrite levels. Our results have showed that mercury levels were significant in all three glands compared to the respective control. It also showed lower levels of ACAP, as well as higher LPO and nitrite levels. The proteomic profile presented impairments on structural components of cytoskeleton, metabolic pathways, and oxidative biochemistry. Thus, the exposure to MeHg was able to generate oxidative stress that could be associated with changes in the proteomic profile of salivary glands.

## 1. Introduction

Mercury is a high toxic metal with a wide distribution on nature and has been considered as an important health concern [1, 2]. Mercury occurs naturally in the environment on organic and inorganic forms, in which methylmercury (MeHg) and mercury chloride (HgCl_2_) are the most toxic forms, respectively. MeHg is an alkylmercury compound that is derived from the methylation of Hg^+2^ by methanogenic and sulfate-reducing bacteria on aquatic ecosystems and some soils [3–5].

The anthropogenic action leads to higher concentrations on biosphere and may expose animals of aquatic food chain to damage [5]. Many researches have reported that fishes and other seafood are the main sources of human contamination by low daily doses, in which about 90% of Hg is present on its organic form, leading to higher bioaccumulation and biomagnification [6, 7].

Hg also has been described as a huge pollutant able to accumulate and induce cellular impairments in organs related to metabolism and excretion, as the liver and kidneys [8–11]. Few studies [12–14] have demonstrated the effects of HgCl_2_ in salivary glands, and there is no evidence of MeHg effects. In this way, it is important to describe the MeHg-induced alteration in salivary glands to elucidate the consequences of this metal exposure.

The parotid, submandibular, and sublingual are the main salivary glands, representing about 90% of the total salivary human production. Salivary glands are important organs on the maintenance of oral cavity homeostasis; their major function is salivary secretion that is responsible for balancing the microbiota, pH, and carbohydrates catalysis in the mouth [15, 16]. Moreover, studies of metal toxicological effects on salivary glands are still rare, especially about MeHg. The aim of this study was to evaluate whether the chronic exposure to low doses of MeHg is able to impair biochemical parameters and the proteomic profile, which could be related to dysfunctions in the organs.

## 2. Materials and Methods

### 2.1. Animals and Experimental Groups

A total of 40 male Wistar rats were obtained from the Federal University of Pará (UFPA, Belém, Brazil), under the BIO 225-14 CEPAE – UFPA, following the NIH Guide for the Care and Use of Laboratory Animals. The animals were maintained in collective cages with 5 animals each and were kept in a climate-controlled room on a 12 h light/dark cycle with water and food ad libitum. Corn oil or MeHg with a daily dose of 0.04 mg/kg was orally administered by gavage over a period of 60 days of exposure according to an adapted protocol from Kong et al. [17]. To regulate the administered dose in animals, they were weekly weighted for adjustment of MeHg concentration and determination of the body mass curve.

### 2.2. Samples Collection

After 24 hours of the last administration, the animals were euthanized by cervical dislocation and had their three pairs of salivary glands (parotid, submandibular, and sublingual) collected and frozen in liquid nitrogen and posteriorly stored in ultrafreezer −80°C until further analysis. One gland from each animal was saved to the assays of oxidative biochemistry, proteomic analysis, and measurement of total mercury deposits.

### 2.3. Mercury Deposits

Total mercury content in the samples was quantified by wet digestion, reduction, and cold vapor atomic absorption spectrometry with gold amalgamation system Mercury Analyzer: SP3D (Nippon Corporation). The estimations were conducted following the protocol previously mentioned by Suzuki et al. [18], in which consists by converting the Hg into elementary Hg vapor in order to quantify in parts per million (ppm) by an absorption cell. The analyses were performed preparing duplicates of each sample of groups, and the values were tabulated to further inferential statistical analysis.

### 2.4. Oxidative Biochemistry Analysis

The samples were first thawed and homogenized in 700 *μ*L Tris-HCl buffer (20 mM, pH 7.4). A volume of 200 *μ*L from the total homogenate was used to perform the measurement of antioxidant capacity against peroxyl radicals (ACAP), whereas 300 *μ*L was used to measure nitrite levels and 100 *μ*L to lipid peroxidation assay (LPO).

First, ACAP was measured as previously described by Amado et al. [19]. The supernatant of each sample after centrifugation was, in triplicate, exposed to a peroxyl radical generator, the 2,2′-azobis 2 methylpropionamidine dihydrochloride (ABAP; 4 mM; Aldrich), and, in another triplicate, they received only ultrapure water in a 96-well microplate. After reaction, the fluorescence generated was measured in a fluorimeter (Victor X3, Perkin Elmer) at 35°C. After the first reading for determination of background fluorescence values, a total of 10 *μ*l of 2′,7′ (H_2_DCF-DA) 40 nM was added to the microplate, with a reading in every 5 minutes during 60 minutes [19, 20]. The results were expressed according to the difference between areas below the curves created by total fluorescence production of samples with and without ABAP after application to a second order polynomial function, being named as a relative area. High-relative areas mean low antioxidant activity, once the sample had low capacity to neutralize the peroxyl radicals. Results were expressed as percentages of control group.

The remaining homogenate was centrifuged at 21.000*g* for 20 minutes, and the supernatant was used to perform the assays. The concentration of nitrite was determined following the protocol previously adapted by Fagundes et al. [21], which is based on a reaction with Griess reagent (0.1% N-(1-naphthyl) ethylenediaminedihydrochloride; 1% sulfanilamide in 5% phosphoric acid; 1 : 1) proceeding the absorbance measurement on a spectrophotometer at 550 nm, and compared to standard solutions of sodium nitrite.

The level of LPO was established by measurement of malonaldehyde (MDA) levels based on Esterbauer and Cheeseman [22] method adapted by Fagundes et al. [21]. An aliquot of the supernatant was processed as described by the Bioxytech LPO-568 kit (Cayman Chemical). This kit is a chromogenic reagent that reacts with MDA at 45°C. The absorbance measurement on spectrophotometers was performed at 586 m wavelength.

The total protein content in the supernatants (20 *μ*L) was analyzed by Bradford [23] method. After the correction for protein concentration, results of lipid peroxidation and nitrite levels were expressed in percentages of control groups.

### 2.5. Proteomic

The frozen salivary glands were homogenized in a cryogenic mill (model 6770, Spex, Metuchen, NJ, EUA). For protein extraction, gland homogenate was incubated in lysis buffer (7 M urea, 2 M thiourea, 4% CHAPS, 1% IPG buffer pH 3–10, and 40 mM DTT) for 1 hour at 4°C with continuous shaking. In order to recovery the soluble proteins and the supernatant, the homogenate was centrifuged at 15.000 rpm for 30 minutes at 4°C. The proteins were precipitated using the kit PlusOne 2D Cleanup (GE Healthcare, Uppsala, Sweden), as recommended by the manufacturer. Pellets were resuspended in rehydration buffer (7 M urea, 2 M thiourea, 0.5% CHAPS, 0.5% IPG buffer pH 3–10, 18 mM DTT, and 0.002% bromophenol blue). Twenty-five *μ*L of salivary gland proteins from each animal of the same group was combined to constitute a pool that was centrifuged for clarification. To each pool, 50 mM AMBIC containing 3 M urea was added. Each sample was filtered twice in 3 kDa AMICON (Millipore, St. Charles, MO, USA). The protein content was measured in the pooled samples by Bradford protein assay [23]. To each sample (50 *μ*g of total protein for each pool in a volume of 50 *μ*L), 10 *μ*L of 50 mM AMBIC was added. In sequence, 25 *μ*L of 0.2% RapiGEST™ (Waters Co., Manchester, UK) was added and incubated at 80°C for 15 min. Following, 2.5 *μ*L of 100 mM DTT was added and incubated at 60°C for 30 min. Also, 2.5 *μ*L of 300 mM IAA was added and incubated for 30 min at room temperature (under dark). Then, 10 *μ*L of trypsin (100 ng; Trypsin Gold Mass Spectrometry, Promega, Madison, USA) was added and digestion occurred for 14 h at 37°C. After digestion, 10 *μ*l of 5% TFA was added, incubated for 90 min at 37°C, and the sample was centrifuged (14,000 rpm for 30 min). The supernatant was collected, and 5 *μ*L of ADH (1 pmol/*μ*L) plus 85 *μ*L 3% ACN was added.

After protein extraction is completed, separation and identification of peptides were performed on a nanoAcquity UPLC-Xevo QTof MS system (Waters, Manchester, UK). The difference in expression among the groups was obtained using PLGS software, considering *p* < 0.05 for downregulated proteins and *p* > 0.95 for upregulated proteins. The bioinformatics analysis was performed using Uniprot protein ID accession numbers to map their associated encoding Uniprot gene entries for the comparison of control × MeHg. Gene ontology (GO) annotation of biological process was performed using Cytoscape v3.0 software, with the Cluego v2.0.7 plugin. Uniprot IDs were uploaded to the software and analyzed with default parameters, which specify an enrichment (right-sided hypergeometric test) correction method using Bonferroni step down, analysis mode “Function” and load gene cluster list for *Rattus novergicus*, evidence codes “All,” set networking specificity “medium” (GO levels 3 to 8), and KappaScoreThreshold 0.4. The protein-protein interaction (ppi) network was built by ClusterMarker, another Cytoscape plugin. After the network creation, we edited nodes and edge colors according to each group.

### 2.6. Statistical Analysis

Statistical comparison of body weight gains between control and MeHg groups was performed using one-way analysis of variance (ANOVA).

All values obtained from the biochemical and total mercury quantification analysis were plotted on GraphPad Prism 5.0 software (San Diego, CA, USA) and were expressed, respectively, as percentage of control and mean ± SEM. All data were compared using Student's *t*-test with a level of significance of *p* < 0.05.

## 3. Results

### 3.1. Body Weight Measurement and Deposits of Total Hg in Salivary Glands

**Figure 1:**
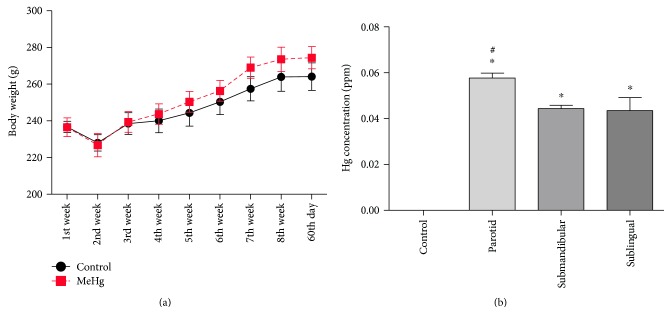
(a) Effects of MeHg chronic exposure at a dose of 0.04 mg/kg/day during 60 days on body mass gain of rats. The results are expressed as mean ± SEM (*n* = 20 animals per group) with one-way ANOVA with repeated measures followed by Tukey test. (b) Total mercury deposits on salivary glands of rats exposed to MeHg for 60 days. The values are expressed as mean ± SEM of Hg concentrations. ^∗^*p* < 0.05 compared to control group and # compared to others glands (one-way ANOVA and Tukey posttest).

Although a normal increase on body mass over the 60 days of MeHg exposure was observed, no difference was observed at the final weight (Figure 1(a)). The MeHg chronic exposure was able to promote Hg deposits (ppm) on salivary glands (Figure 1(b)). Our results showed, after the 60th day of MeHg exposure, a significantly Hg deposit in all three types of rat glands compared to the control groups. We also showed a higher deposit on parotid, suggesting a major tropism than submandibular and sublingual glands.

### 3.2. Chronic Exposure of MeHg Impairs the Oxidative Biochemistry

**Figure 2:**
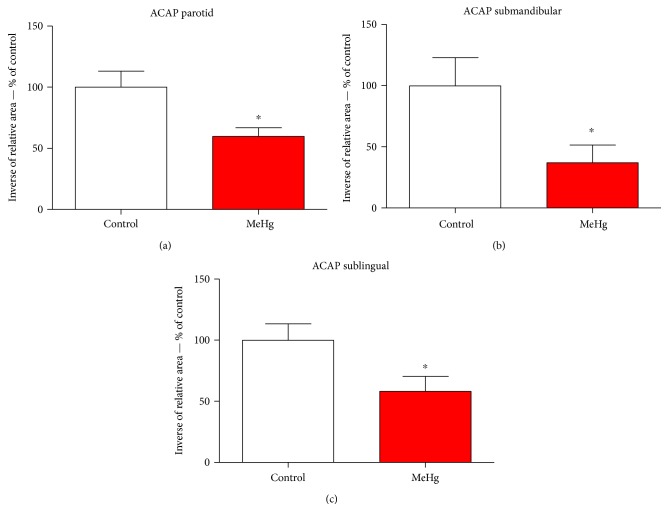
Levels of antioxidant capacity against peroxyl radical parotid (a), submandibular (b), and sublingual salivary glands (c) of animals exposed chronically to methylmercury. The values are expressed as percentage of control ± SEM. ^∗^*p* < 0.05 (Student's *t*-test).

In order to analyze the possible involvement of cellular stress, we performed oxidative/nitrosative biochemistry assay. Our data suggest that MeHg is able to induce impairment at the antioxidant defense system by reducing the total antioxidant capacity. Results showed lower ACAP levels in submandibular (Figure 2(c)) showing 36.92% ± 14.51 more RNS than in control group, whereas sublingual (Figure 2(b)) showed 58.24% ± 12.26 and parotid (Figure 2(a)) 59.67% ± 7.32 more than control, respectively.

**Figure 3:**
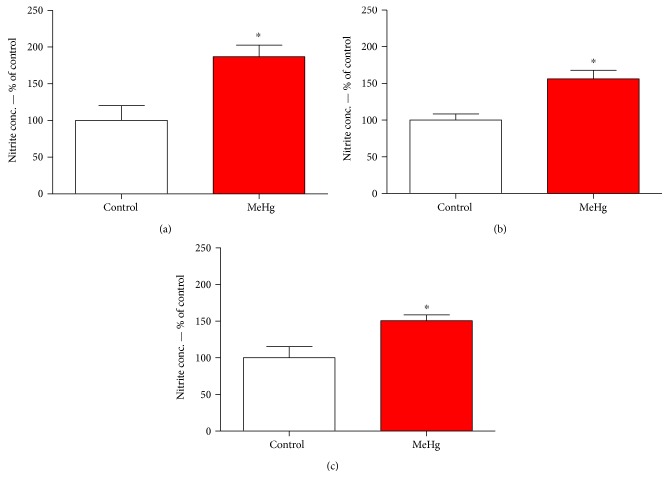
Levels of nitrite concentration in parotid (a), submandibular (b), and sublingual salivary glands (c) of animals exposed chronically to methylmercury. The values are expressed as percentage of control ± SEM. ^∗^*p* < 0.05 (Student's *t*-test).

Our result showed that nitrite concentrations are higher at all three types of rat salivary gland than at the control group. The results showed higher levels in parotid (Figure 3(a)) showing 86.89% ± 15.71 more RNS than in control group, whereas submandibular (Figure 3(b)) showed 56.42% ± 11.65 and sublingual (Figure 3(c)) 50.45% ± 8.09 more than the control, respectively.

**Figure 4:**
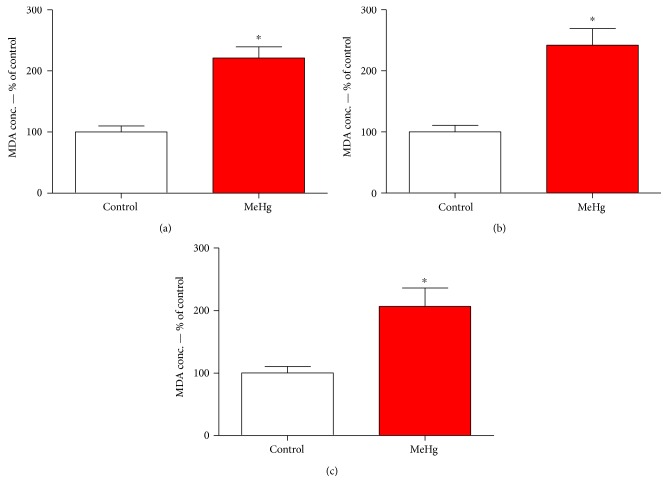
Levels of lipid peroxidation in parotid (a), submandibular (b), and sublingual (c) glands of animals exposed chronically to methylmercury. The values are expressed as percentage of control ± SEM. ^∗^*p* < 0.05 (Student's *t*-test).

We further showed that MeHg is able to induce lipid peroxidation, by the yields of MDA (Figure 4), on salivary glands of MeHg group. The results showed higher levels of MDA in all three major glands. All glands of MeHg group showed 100% more damaged than those of the control animals. The parotid (Figure 4(a)) showed about 121.16% ± 18.2 higher, submandibular (Figure 4(b)) about 141.94% ± 27.18 higher, and sublingual (Figure 4(c)) about 106.59% ± 29.38 more when compared to the control.

### 3.3. MeHg Changes the Proteomic Profile of Rat Salivary Glands

This exposure model revealed a total of 15 proteins downregulated and 22 proteins upregulated on parotid glands (Table 1); 6 proteins downregulated and 7 upregulated on submandibular glands (Table 2); and 7 proteins downregulated and 2 upregulated on sublingual glands (Table 3). The proteomic analysis of the three salivary glands also revealed several proteins that were only found in one of the groups, being absent in the other (Supplementary Tables available online at https://doi.org/10.1155/2017/5653291).

**Figure 5:**
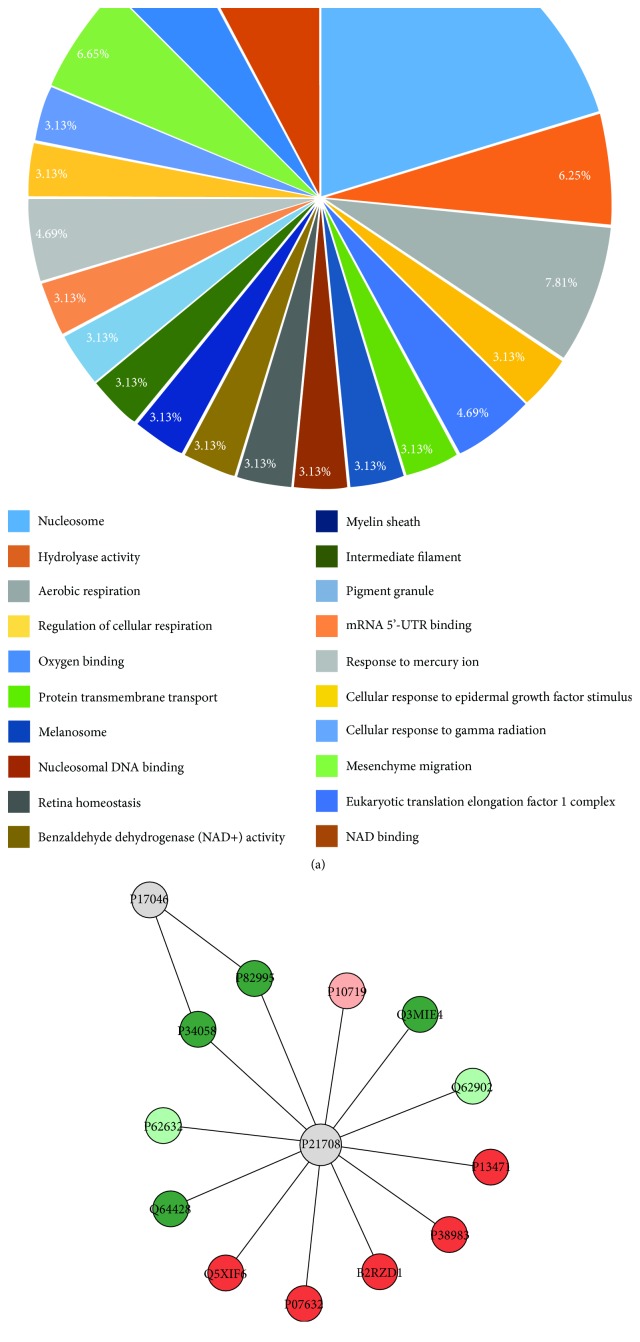
Functional distribution of proteins identified with differential expression (a) in rat parotid glands exposed to MeHg versus control group. Categories of proteins based on GO annotation biological process, molecular function, and cellular component. Terms significant (Kappa = 0.4) and distribution according to percentage of number of genes association. And subnetwork (b) created by ClusterMarker app to establish the interaction among identified proteins of parotid glands with differentiated expression on exposed group when compared to the control group. The node colors indicate the differential expression of respective protein named with its accession ID. Red and dark green indicate proteins found in the control and exposed groups, respectively. Light green and pink indicate upregulated and downregulated proteins, respectively. Gray nodes represent those proteins that were not identified on this study, but they interacted on the network. The access identification number in nodes corresponds to P17046: lysosome-associated membrane glycoprotein 2, P82995: heat shock protein HSP 90-alpha, P34058: heat shock protein HSP 90-beta, P21708: mitogen-activated protein kinase 3, P10719: synaptic vesicle membrane protein VAT-1 homolog, Q3MIE4: protein ERGIC-53, Q62902: protein ERGIC-53, P13471: 40S ribosomal protein S14, P38983: 40S ribosomal protein SA, B2RZD1: protein Sec61b, P07632: superoxide dismutase (cu-Zn), Q5XIF6: tubulin alpha-4A chain, Q64428: trifunctional enzyme subunit alpha, mitochondrial, and P62632: elongation factor 1-alpha 2.

In parotid gland, MeHg was able to change 20 categories of proteins classified by Cytoscape according to GO (Figure 5(a)). Our data showed that they are related to the following biological processes: nucleosome (20.31%), aerobic respiration process (7.81%), NAD binding (7.81%), mesenchyme migration (6.65%), hydrolyase activity (6.25%), oxygen binding (4.69%), and response to mercury ion (4.69%). Regulation of cellular respiration, protein transmembrane transport, melanosome, nucleosomal DNA binding, retina homeostasis, benzaldehyde dehydrogenase (NAD+) activity, myelin sheath, intermediate filament, pigment granule, mRNA 5′-UTR biding, cellular response to epidermal growth factor stimulus, and cellular response to gamma radiation corresponded to 3.13% each.

The ppi network of parotid gland (Figure 5(b)) showed a central protein (mitogen-activated protein kinase 3; P21708), which interacts with proteins related to stress response (heat shock protein HSP 90-alpha, P82995; heat shock protein HSP 90-beta, P34058), molecular function proteins (synaptic vesicle membrane protein VAT-1 homolog, Q3MIE4; protein ERGIC-53, Q62902; 40S ribosomal protein S14, P13471; 40S ribosomal protein AS, P38983; Sec61 beta subunit, B2RZD1; elongation factor 1-alpha 2, P62632), oxidative stress enzymes (superoxide dismutase (Cu-Zn), P07632), microtubule component (tubulin alpha-4A chain, Q5XIF6), and energetic metabolism process (trifunctional enzyme subunit alpha, mitochondrial, Q64428; ATP synthase subunit beta, mitochondrial, P10719).

**Figure 6:**
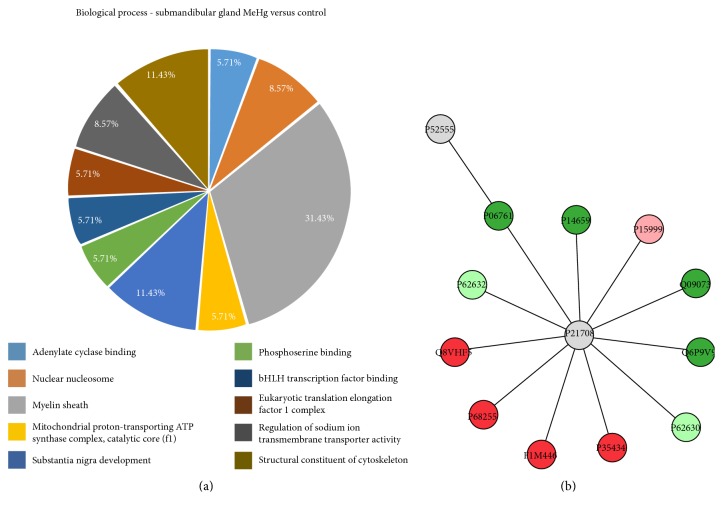
Functional distribution of proteins (a) identified with differential expression in rat submandibular glands exposed to MeHg versus control group. Categories of proteins based on GO annotation biological process, molecular function, and cellular component. Terms significant (Kappa = 0.4) and distribution according to percentage of number of gene association. Subnetwork (b) created by ClusterMarker app to establish the interaction among identified proteins of submandibular glands with differentiated expression on exposed group when compared to control group. The node colors indicate the differential expression of respective protein named with its accession ID. Red and dark green indicate proteins found in control and exposed groups, respectively. Light green and pink indicate upregulated and downregulated proteins, respectively. Gray nodes represent those proteins that were not identified on this study, but they interacted on the network. The access identification number in nodes corresponds to P52555: endoplasmic reticulum resident protein 29, P06761: 78 kDa glucose-regulated protein, P14659: heat shock-related 70 kDa protein 2, P15999: ATP synthase subunit alpha, mitochondrial, Q09073: ADP/ATP translocase 2, Q6P9V9: tubulin alpha-1B chain, P62632: elongation factor 1-alpha 2, P35434: ATP synthase subunit delta, mitochondrial, F1M446 protein RGD1306148, P68255: 14-3-3 protein theta, Q8VHF5: citrate synthase, mitochondrial, and P62632: elongation factor 1-alpha 2.

As for the submandibular gland, our results showed that 10 categories of proteins classified by Cytoscape, according to GO, were significantly altered upon chronic exposure to MeHg (Figure 6(a)). The myelin sheath proteins correspond to 31.43%; the structural constituent of cytoskeleton and the substance nigra development represent 11.43%, each. Moreover, the nuclear nucleosome represents 8.57% and regulation of sodium ion transmembrane transporter activity symbolizes 8.57% of the proteins with alterations. Adenylate cyclase binding, mitochondrial proton-transporting ATP synthase complex, catalytic core F (1), phosphoserine binding, bHLH transcription factor binding, and eukaryotic translation elongation factor 1 complex corresponded to 3.13% each.

Similarly to what was found for the parotid gland, most of the proteins with altered expression in the submandibular gland interacted with mitogen-activated protein kinase 3 (P21708), which was found in the center of the ppi network. The interacting proteins were those related to stress response (P14659), energetic metabolic process (ATP synthase subunit alpha, mitochondrial, P15999; ADP/ATP translocase 2, Q09073; ATP synthase subunit delta, mitochondrial, P35434 and citrate synthase, mitochondrial, Q8VHF5), microtubule constituent (tubulin alpha-1B chain, Q6P9V9), molecular functions (elongation factor 1-alpha 2, P62632; uncharacterized protein, F1M446; 14-3-3 protein theta, P68255; elongation factor 1-alpha 2, P62632), and cellular component (endoplasmic reticulum resident protein 29, P52555; 78 kDa glucose-regulated protein, P06761) (Figure 6(b)).

**Figure 7:**
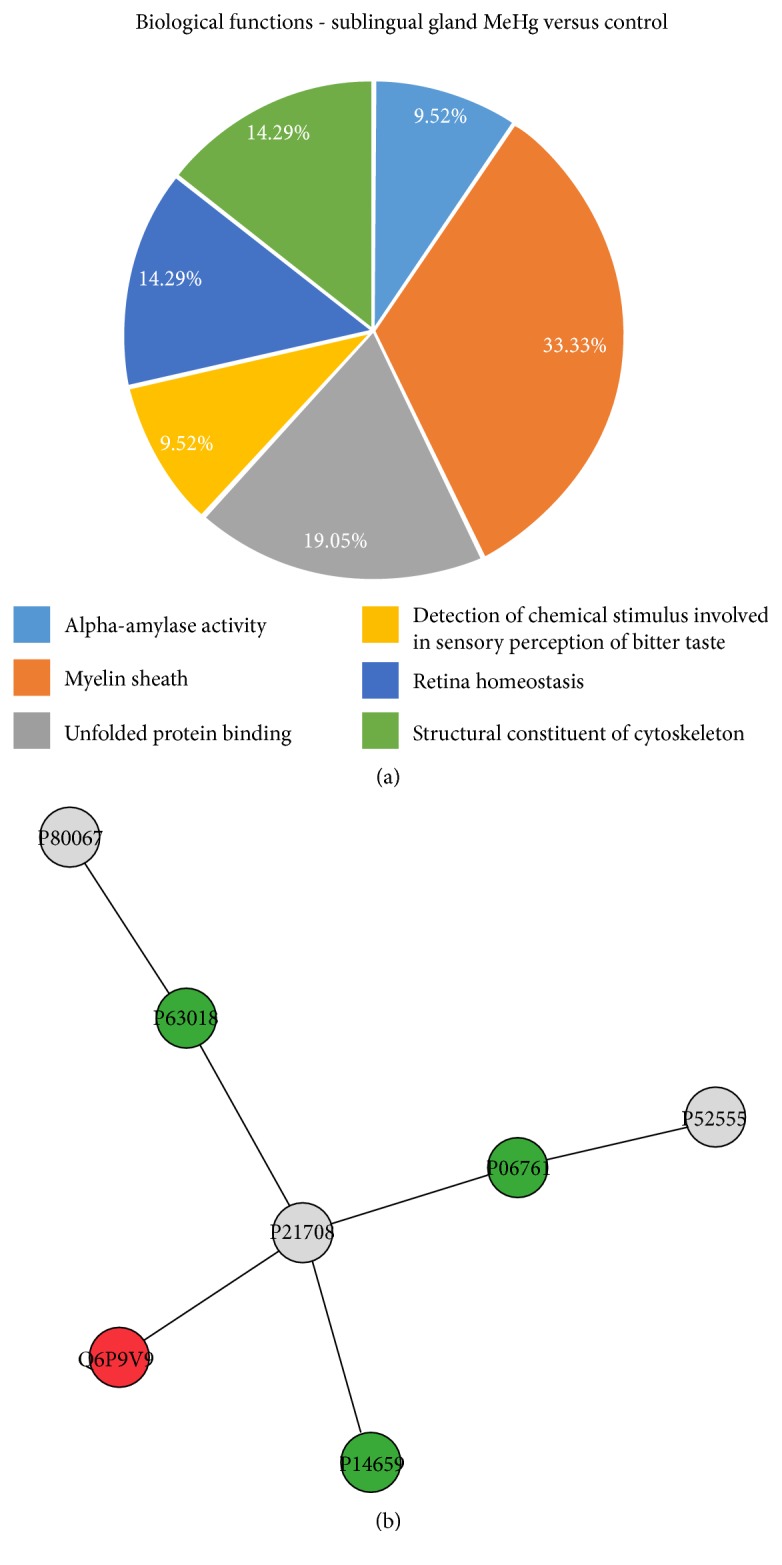
Functional distribution of proteins (a) identified with differential expression in rat submandibular glands exposed to MeHg versus control group. Categories of proteins based on GO annotation biological process, molecular function, and cellular component. Terms significant (Kappa = 0.4) and distribution according to percentage of number of gene association. Subnetwork (b) created by ClusterMarker app to establish the interaction among identified proteins of sublingual glands with differentiated expression on exposed group when compared to control group. The node colors indicate the differential expression of respective protein named with its accession ID. Red and dark green indicate proteins found in control and exposed groups, respectively. Light green and pink indicate upregulated and downregulated proteins, respectively. Gray nodes represent those proteins which were not identified on this study, but they interacted on the network. The access identification number in nodes corresponds to P80067: dipeptidyl peptidase 1, P63018: heat shock cognate 71 kDa protein, P21708: mitogen-activated protein kinase 3, Q6P9V9: tubulin alpha-1B chain, P14659: heat shock-related 70 kDa protein 2, P06761: 78 kDa glucose-regulated protein, P52555: endoplasmic reticulum resident protein 29.

Sublingual glands presented the lower number of protein groups identified on Cytoscape, following ClueGO app GO classification (Figure 7(a)). Each of the 6 groups of proteins is represented by myelin sheath (33.33%), unfolded protein binding (19.05%), retina homeostasis (14.29%), structural constituent of cytoskeleton (14.29%), detection of chemical stimulus involved in sensory perception of bitter taste (9.52%), and alpha-amylase activity (9.52%).

The ppi network of the sublingual gland (Figure 7(b)) showed the same protein in the center of the network (mitogen-activated protein kinase 3; P21708), which the proteins with altered expression interacted. The interaction proteins were related mainly to biological processes, such as mitosis and cytoskeletal rearrangements. In addition, proteins associated to stress response (heat shock cognate 71 kDa protein, P63018 and heat shock-related 70 kDa protein 2, P14659) and microtubule constituent (tubulin alpha-1B chain, Q6P9V9) were clustered by ClusterMarker app.

## 4. Discussion

In this study, we showed for the first time that MeHg, in a chronic period, causes damage to the salivary gland proteomic profile and oxidative stress. A significant total Hg deposit in the gland tissue structure was verified, as well as a membrane cell damage by higher LPO levels, nitrogen species formation due to oxidative stress, and low antioxidant capacity against peroxyl radicals. In addition, the novelty of the toxicological evaluation on the proteomic profile of rat glands after MeHg exposure brings the association with differences in expression at important proteins of metabolic pathways, oxidative biochemistry, and mainly in the composition of cytoarchitecture.

Most of the studies about MeHg toxicity are under acute exposures and with high MeHg concentrations, even though the reality faced by the population of endemic regions of this metal intoxication is different. Recent studies showed that long-term intoxication includes other effects such as cardiovascular diseases and genotoxicity [24–27]. This latter one includes oxidative stress and alterations of cytoskeleton proteins as main causes for these effects. The known effects of MeHg are mainly given by chronic exposures, thus, the model proposed by Kong et al. [17], in which we based our study, mimics a daily and continuous exposure in low doses. The exposure time of our study brings the possibility to find interesting results on an oxidative biochemistry and proteomic profiles at rat glands, showing that even at low doses, a chronic MeHg exposure is able to induce molecular and cellular damages in living organisms.

For understanding, the nomination of low dose used in this researcher is important to consider other anatomic regions and the toxic effects. The lowest observed adverse effect level (LOAEL) for neurotoxic effects such as paresthesia is 50 ppm of mercury in hair [28]. This level in hair corresponds approximately to 1 ppm of mercury in brain, according to the proportion of 250 : 50 : 1 for hair : brain : whole blood contents (reviewed by Branco et al. [29]).

The mercury content in brains of rats exposed to the dose used in our work (0.04 mg/kg per day for 8 weeks) is about a half of that level, that is, about 0.5 ppm [17, 30], characterizing a low exposure. Moreover, animals exposed to this low level of methylmercury did not develop neurotoxic effects such as alterations of forelimb grip strength, running wheel performance, or hind limb cross [30], supporting that this mercury level in the brain is not sufficient to cause deleterious neurobehavioral consequences.

Looking at Figure 1, an interesting fact is that salivary glands accumulated about 10 times less mercury than 0.5 ppm. However, our results show for the first time that this low dose is sufficient to cause significant oxidative stress and alterations of protein regulation in salivary glands, revealing the high susceptibility of this tissue to the toxic effects of methylmercury.

The main form of MeHg intoxication is the oral through food [6], so the preferential site of absorption is in the intestinal lumen by enterocytes. The experimental model of intragastric gavage, used in this work, illustrates the MeHg intake by food consumption. MeHg is formed by a methyl group attached to the Hg atom (CH_3_Hg), and this allows a greater affinity with thiol groups, mainly of protein components [31].

The MeHg gains systemic circulation from the efflux through the basolateral membrane of enterocytes and on this part of membrane surface. There are multidrug resistance-associated proteins (MRPs), neutral amino acid transporters, as LAT2, and organic anions transporter (OAT) [32]; then, it is suggested that these proteins' action is related to MeHg efflux. Moreover, some MRPs need GSH as cosubstrate for their effectiveness, so MeHg may also undergo efflux to systemic blood circulation. According to Martinez-Madrigal and Micheau [33], blood vessels as external carotid, sublingual, submental, and facial supply all glands, suggesting that vascularization is the main path by which Hg forms deposits by intragastric gavage intoxication.

In this work, we found significant Hg concentration (ppm) in the three main salivary glands in comparison to the control group. Studies about the proteomic profile of salivary glands have suggested that parotid is responsible to produce the major part of the protein components of saliva [34–36], and this could be the reason to a higher absorption to this gland showed by our group, once this metal has an affinity to structures present in many proteins.

Studies appoint that MeHg induces multiple effects within cell homeostasis. MeHg is able to induce cell death [37], impairment on cell proliferation and differentiation [38], disorganization of cytoskeleton [39, 40], DNA mutation [41], impairments on transcriptome [42], and proteome [43]. In addition, according to the literature, these damages on biological processes and structures could be associated with oxidative/nitrosative biochemistry misbalance due to oxidative stress.

Cellular membranes are crucial targets of MeHg-induced toxicity, and due to its constitution of unsaturated lipids, the products MDA and 4-HNE are the most mutagenic and toxic products of LPO (for review see Ayala et al. [44]); in addition, organelle membranes also may suffer due to LPO [45]. In this study, we showed an increase on MDA levels in rat salivary glands after MeHg exposure, suggesting a high susceptibility of glandular tissue to oxidative/nitrosative stress.

The protein peroxidation products, that is, peroxyl radical, are highly harmful to cell membranes. Beyond the importance of stress in the glandular homeostasis after MeHg exposure, the production of peroxyl radical seems to be an important linkage between lipid peroxidation and protein oxidation, once peroxyl could be an initiator factor of LPO [46–48]. This way, our results suggest that MeHg chronic exposure can decrease the ACAP in salivary glands and lead the glandular stress, seen by nitrite and MDA levels, which may lead to damage at membrane lipid and to cellular proteins.

It is well known that oxidative/nitrosative stress is able to impair gene expression [49] and protein translation [50] driving to oxidized and nonfunctional proteins. In this way, we further characterized for the first time the proteomic changes under oxidative stress after MeHg exposure on salivary rat glands. The global proteomic results of proteins up- and downregulated in parotid, submandibular, and sublingual glands are described in Tables 1, 2, and 3, respectively.

The literature has pointed that the imbalance in oxidative stress may lead to disorders on cellular pathways of energetic metabolism, such as the heat shock class of proteins [51–53]. We showed that in parotid glands, MeHg was able to induce downexpression of heat shock cognate 71 proteins (D4A4S3 and M0R8M9) and induce exclusively the expression of heat shock proteins (HSP) 90 (P82995 and P34058) in MeHg group. In submandibular, we also found heat shock proteins (M0R660 and P14659) that were unique in MeHg group, while in the sublingual gland, we found the exclusive expression of heat shock cognate 71 (P63018) and heat shock-related 70 (P14659) and expression of heat shock protein beta-6 (P97541) only in the control group. HSPs have been already proposed as new therapeutic tools for some disorders as cancer and cardiovascular diseases, with a protective role especially for HSP90 [54]. The results of the present study support a main role for this protein also in methylmercury intoxication.

In addition, glutathione S-transferase P (P04906) and components of cytochrome (Q62737 and Q68FY0) were exclusively expressed in parotid control group, suggesting an important association on protein changes and MeHg-induced oxidative stress.

The metabolic pathway class of proteins was also changed after MeHg exposure. In parotid, the proteins ATP synthase subunit alpha (F1LP05) and ATP synthase subunit beta, mitocondrial (P10719), were downregulated and malate dehydrogenase, cytoplasmic (O88989), and aconitate hydratase (G3V6S2) were unique in the control group. In submandibular, ATP synthase subunit alpha, mitochondrial (P15999), was downregulated, while ADP/ATP translocase 2 (Q09073), ATP synthase subunit delta, mitochondrial (P35434), citrate synthase (G3V936), citrate synthase, and mitochondrial (Q8VHF5) were present only in the control glands. Our results corroborate with metabolic pathway disorders established in others experimental models [17, 55].

MeHg treatment also impaired negatively the proteins of cellular components, such as cytoskeleton, from the main constituents of salivary glands. We have showed that the proteins related to muscle cell contraction (actin, alpha skeletal muscle, P68136; actin, aortic smooth muscle, P62738; and actin, gamma-enteric smooth muscle, P63269), in parotid glands were downregulated after MeHg exposure, which may suggest possible damages in myoepithelial cells present in the glands. We also showed downregulation of keratin, type II cytoskeletal 8 (Q10758) that is an important component of conjunctive tissue.

Concerning to microtubule constitution, we showed here that tubulin beta chain (B4F7C2) and tubulin beta-4B chain (Q6P9T8) were downregulated, corroborating with the literature, which suggests that microtubule depolymerization is one of the mechanisms of Hg damage on living cells [25, 56, 57].

A collagen chain protein (collagen alpha-1(I) chain, P02454), cytokeratins (keratin, type I cytoskeletal 10, Q6IFW6; keratin, type I cytoskeletal 14, Q6IFV1; keratin, type I cytoskeletal 15, Q6IFV3; keratin, type I cytoskeletal 17, Q6IFU8; keratin, type I cytoskeletal 24, Q6IFX1; keratin, type I cytoskeletal 42, Q6IFU7; and keratin, type II cytoskeletal 7, Q6IG12), and tubulin chains (tubulin beta chain, Q4QQV0 and tubulin alpha-4A chain, Q5XIF6) were found exclusively in control parotid glands. In sublingual glands, we also found conjunctive constituent (collagen alpha-2(I) chain, P02466), cytoskeleton (Q5BJY9) and microtubule (tubulin alpha-1A chain, P68370; tubulin alpha-1B chain, Q6P9V9; and tubulin alpha-1C chain, Q6AYZ1) components uniquely in the nonexposed group.

As previously mentioned, salivary glands are responsible for salivary formation and flow; this way, the acinar and tubular constituents have great importance in physiology. In the proteomic analysis, downregulation of alpha-amylase (E9PSQ1) was observed, as well as prolactin-induced protein, isoform CRA_d (G3V812), and prolactin-inducible protein homolog (O70417) in the parotid. An interesting protein related to biomineralization (submandibular gland secretory Glx-rich protein CB, P08462) was upregulated in the submandibular of the exposed group. Salivary flow is first derived by ionic exchanges by sodium/potassium bombs in acini and intercalated portions of secretory duct system [58], and in this study, we noticed that sodium/potassium-transporting ATPase subunit alpha-1 (P06685) was exclusive of control group in sublingual gland, similarly to sodium channel subunit beta-3 (Q9JK00) and sodium/potassium-transporting ATPase subunit alpha-2 (P06686) that were unique of submandibular gland of animals not submitted to MeHg exposure.

The proteomic profile changes shown by this work suggest that MeHg exposure leads to salivary gland damage, since several proteins were shown to be affected, compromising essential biological functions and components that under normal circumstances are fundamental do cell and tissue viability.

## 5. Conclusion

The proteomic profile verified after MeHg exposure in this study allowed us to see what is underlying the oxidative stress effects in salivary glands. Our findings have showed that salivary glands are also targets for MeHg in systemic intoxication, at low doses and chronic exposure, being able of triggering imbalance in oxidative biochemistry and, consequently, the modulation of proteomic profile of the glands.

## Supplementary Material

Supplementary Table 1. Proteins exclusively present in rat parotid glands in MeHg (Hg) group. Supplementary Table 2. Proteins exclusively present in rat parotid glands control group. Supplementary Table 3. Proteins exclusively present in rats submandibular glands in Hg (MeHg) group. Supplementary Table 4. Proteins exclusively present in rats submandibular glands in control group. Supplementary Table 5. Proteins exclusively present in rats sublingual glands in Hg (MeHg) group. Supplementary Table 6. Proteins exclusively present in rats sublingual glands in control group.

## Figures and Tables

**Table 1:** Identified proteins with expression significantly altered in rat parotid glands of group exposed to Mehg (Hg).

^a^Access number	Protein name description	PLGS score	Fold change
			Hg
P68136	Actin, alpha skeletal muscle	1873.1	−0.9
P62738	Actin, aortic smooth muscle	1713.4	−0.9
P63269	Actin, gamma-enteric smooth muscle	1713.4	−0.9
F1LP05	ATP synthase subunit alpha	548.16	−0.8
P10719	ATP synthase subunit beta, mitochondrial	353.85	−0.8
P21704	Deoxyribonuclease-1	268.66	−0.6
D3ZXS6	Elongation factor 1-alpha	327	2.4
M0R757	Elongation factor 1-alpha	343.66	2.5
P62630	Elongation factor 1-alpha 1	343.66	2.5
P62632	Elongation factor 1-alpha 2	327	2.5
D4A4S3	Heat shock cognate 71 kDa protein	151.3	−0.7
M0R8M9	Heat shock cognate 71 kDa protein	739.22	−0.8
P01946	Hemoglobin subunit alpha-1/2	1975.8	1.2
P02091	Hemoglobin subunit beta-1	1841.8	1.8
D3ZWE0	Histone H2A	764.11	2.0
P02262	Histone H2A type 1	764.11	1.9
P0C169	Histone H2A type 1-C	764.11	1.8
P0C170	Histone H2A type 1-E	764.11	2.0
Q64598	Histone H2A type 1-F	764.11	1.9
P0CC09	Histone H2A type 2-A	764.11	1.8
Q4FZT6	Histone H2A type 3	764.11	1.8
Q00728	Histone H2A type 4	764.11	1.7
A9UMV8	Histone H2A.J	764.11	1.9
P0C0S7	Histone H2A.Z	764.11	1.9
D3ZK97	Histone H3	258.66	1.2
P62804	Histone H4	2231.9	1.3
Q10758	Keratin, type II cytoskeletal 8	863.19	−0.8
D3ZUQ1	Lipase	396.87	1.3
G3V812	Prolactin induced protein, isoform CRA_d	7686.8	−0.9
O70417	Prolactin-inducible protein homolog	7084.7	−0.9
F1LRA1	Protein ERGIC-53	400.1	1.2
F1M6C2	Protein LOC103691939	343.66	2.5
F1LZI1	Protein LOC680121	538.95	−0.7
P02770	Serum albumin	489.45	1.3
B4F7C2	Tubulin beta-4	455.5	−0.7
Q6P9T8	Tubulin beta-4B chain	455.5	−0.7
M0RCB1	Uncharacterized protein	769.68	−0.8

The identified proteins are organized according to the alphabetical order. Relative differential expression is indicated by positive value, when the protein is upregulated, and by negative values (−), when the protein is downregulated in the comparison between groups. ^a^Identification is based on protein ID from UniProt protein database (http://www.uniprot.org/).

**Table 2:** Identified proteins with expression significantly altered in rats' submandibular glands of group exposed to Mehg (Hg).

^a^Access number	Protein name description	PLGS score	Fold change
			Hg
Q08163	Adenylyl cyclase-associated protein 1	148.55	−0.2
P15999	ATP synthase subunit alpha, mitochondrial	560.23	−0.6
M0R757	Elongation factor 1-alpha	225.49	1.6
P62630	Elongation factor 1-alpha 1	225.49	1.6
P62632	Elongation factor 1-alpha 2	188.54	1.8
O88752	Epsilon 1 globin	2060.91	−0.3
P11517	Hemoglobin subunit beta-2	2099.64	−0.3
D3ZNH4	Histone H2B	3909.79	−0.7
F1LQ08	Protein Car6	439.43	1.4
F1M6C2	Protein LOC103691939	193.93	1.7
E9PTY1	Protein Prol1	325.88	2.0
P08462	Submandibular gland secretory Glx-rich protein CB	944.25	1.3
D4A5P1	Uncharacterized protein	364.33	−0.6

The identified proteins are organized according to the alphabetical order. Relative differential expression is indicated by positive value, when the protein is upregulated, and by negative values (−), when the protein is downregulated in the comparison between groups. ^a^Identification is based on protein ID from UniProt protein database (http://www.uniprot.org/).

**Table 3:** Identified proteins with expression significantly altered in the rats' sublingual glands of group exposed to MeHg (Hg).

^a^Access number	Protein name description	PLGS score	Fold change
			Hg
E9PSQ1	Alpha-amylase	2696.09	−0.3
F1LPK5	Acidic mammalian chitinase	1384.72	−0.2
G3V812	Prolactin induced protein, isoform CRA_d	6144.39	−0.2
G3V844	Alpha-amylase	344.21	−0.3
M0R7K9	Protein Csap1	1852.75	−0.3
O70417	Prolactin-inducible protein homolog	5900.39	−0.2
P00689	Pancreatic alpha-amylase	344.21	1.7
P02770	Serum albumin	600.4	−0.6
P12020	Cysteine-rich secretory protein 1	221.34	2.5

The identified proteins are organized according to the alphabetical order. Relative differential expression is indicated by positive value, when the protein is upregulated, and by negative values (−), when the protein is downregulated in the comparison between groups. ^a^Identification is based on protein ID from UniProt protein database (http://www.uniprot.org/).
