# Supplementary material for: Oxidative Biochemistry Disbalance and Changes on Proteomic Profile in Salivary Glands of Rats Induced by Chronic Exposure to Methylmercury
Source: Oxid Med Cell Longev. 2017 Jul 24;2017:5653291. doi: 10.1155/2017/5653291 (PMC5546058; doi:10.1155/2017/5653291)
Supplement: Supplementary file 1 — Supplementary Table 1. Proteins exclusively present in rat parotid glands in MeHg (Hg) group. Supplementary Table 2. Proteins exclusively present in rat parotid glands control group. Supplementary Table 3. Proteins exclusively present in rats submandibular glands in Hg (MeHg) group. Supplementary Table 4. Proteins exclusively present in rats submandibular glands in control group. Supplementary Table 5. Proteins exclusively present in rats sublingual glands in Hg (MeHg) group. Supplementary Table 6. Proteins exclusively present in rats sublingual glands in control group. [file 5653291.f1.docx]

Supplementary Table 1. Proteins exclusively present in rat parotid glands in MeHg (Hg) group.

| *^a^*Access number | Protein name description | PLGS Score |
| --- | --- | --- |
| P68511 | 14-3-3 protein eta | 175,01 |
| P61983 | 14-3-3 protein gamma | 117,35 |
| P63102 | 14-3-3 protein zeta/delta | 146,33 |
| Q08163 | Adenylyl cyclase-associated protein 1 | 176,65 |
| D3ZCV5 | Aldehyde dehydrogenase, cytosolic 1 | 130,57 |
| P62161 | Calmodulin | 401,4 |
| G3V9M0 | Cystatin | 650,62 |
| P06214 | Delta-aminolevulinic acid dehydratase | 130,7 |
| Q641Y0 | Dolichyl-diphosphooligosaccharide--protein glycosyltransferase 48 kDa subunit | 271,08 |
| M0RCH0 | Eukaryotic translation initiation factor 3 subunit I | 237,45 |
| B0BNA7 | Eukaryotic translation initiation factor 3 subunit I | 237,45 |
| P82995 | Heat shock protein HSP 90-alpha | 113,76 |
| P34058 | Heat shock protein HSP 90-beta | 216,52 |
| P56574 | Isocitrate dehydrogenase [NADP], mitochondrial | 131,56 |
| P67779 | Prohibitin | 153,08 |
| O88767 | Protein deglycase DJ-1 | 414,81 |
| Q5EBB0 | Protein LOC298795 | 117,35 |
| Q8K3F3 | Protein phosphatase 1 regulatory subunit 14B | 247,45 |
| G3V834 | Protein PRRC1 | 138,28 |
| G3V9A3 | Protein Sfn | 117,35 |
| P51647 | Retinal dehydrogenase 1 | 133,52 |
| Q66X93 | Staphylococcal nuclease domain-containing protein 1 | 112,93 |
| Q3MIE4 | Synaptic vesicle membrane protein VAT-1 homolog | 264,18 |
| Q9JKC9 | Synergin gamma | 116,86 |
| Q64428 | Trifunctional enzyme subunit alpha, mitochondrial | 145,78 |

The identified proteins unique in Hg group are organized according to the alphabetical order. ^a^Identification is based on protein ID from UniProt protein database (<http://www.uniprot.org/>).

Supplementary Table 2. Proteins exclusively present in rat parotid glands control group

| *^a^*Access number | Protein name description | PLGS Score |
| --- | --- | --- |
| P29266 | 3-hydroxyisobutyrate dehydrogenase, mitochondrial | 174,83 |
| P13471 | 40S ribosomal protein S14 | 114,97 |
| P38983 | 40S ribosomal protein SA | 250,76 |
| G3V880 | Aa1017 | 121,63 |
| G3V6S2 | Aconitate hydratase | 95,99 |
| P00330 | Alcohol dehydrogenase 1 | 3123,4 |
| Q8VHE9 | All-trans-retinol 13,14-reductase | 156,89 |
| P49088 | Asparagine synthetase [glutamine-hydrolyzing] | 87,56 |
| Q499T7 | Cilia- and flagella-associated protein 20 | 202,78 |
| P02454 | Collagen alpha-1(I) chain | 93,85 |
| Q62737 | Cytochrome b-245 light chain | 71,92 |
| Q68FY0 | Cytochrome b-c1 complex subunit 1, mitochondrial | 105,14 |
| Q63270 | Cytoplasmic aconitate hydratase | 91,63 |
| Q68FU3 | Electron transfer flavoprotein subunit beta | 131,56 |
| Q68FR9 | Elongation factor 1-delta | 132,39 |
| Q99PF5 | Far upstream element-binding protein 2 | 71,94 |
| P04906 | Glutathione S-transferase P | 558,96 |
| P62828 | GTP-binding nuclear protein Ran | 216,61 |
| Q3KRF2 | High density lipoprotein binding protein (Vigilin) | 110,19 |
| B4F777 | High mobility group nucleosome-binding domain-containing protein 5 | 141,91 |
| M0RCB8 | Histone H3 | 155,2 |
| Q6IFW6 | Keratin, type I cytoskeletal 10 | 119,33 |
| Q6IFV1 | Keratin, type I cytoskeletal 14 | 125,11 |
| Q6IFV3 | Keratin, type I cytoskeletal 15 | 105,3 |
| Q6IFU8 | Keratin, type I cytoskeletal 17 | 122,8 |
| Q6IFX1 | Keratin, type I cytoskeletal 24 | 102,47 |
| Q6IFU7 | Keratin, type I cytoskeletal 42 | 105,3 |
| Q6IG12 | Keratin, type II cytoskeletal 7 | 116,62 |
| O88989 | Malate dehydrogenase, cytoplasmic | 178,2 |
| B1WC26 | N-acetylneuraminic acid synthase | 265,94 |
| G3V8R1 | Nucleobindin 2, isoform CRA_b | 131,59 |
| Q9JI85 | Nucleobindin-2 | 131,59 |
| Q5U317 | Pre-mRNA 3'-end-processing factor FIP1 | 100,68 |
| D4ADC7 | Protein Adgra2 | 75,19 |
| G3V6Y7 | Protein Caap1 | 204,58 |
| F1M5A1 | Protein Calr4 | 127,07 |
| Q63081 | Protein disulfide-isomerase A6 | 142,66 |
| D3ZN42 | Protein Fam122b | 169,96 |
| F1MAF7 | Protein Krt33a | 159,6 |
| Q6IFV6 | Protein Krt35 | 123,6 |
| Q6IFV5 | Protein Krt36 | 102,47 |
| M0RCJ8 | Protein Krt78 | 79,76 |
| F1MAC2 | Protein Krt78 | 79,76 |
| Q6PDV6 | Protein LOC100911847 | 114,97 |
| M0R6R9 | Protein LOC102549011 | 281,46 |
| D3ZL21 | Protein LOC685619 | 93,55 |
| Q6MG75 | Protein Nelfe | 112,12 |
| F1LP37 | Protein Nxnl1 | 75,75 |
| B5DF04 | Protein Rad51 | 116,75 |
| F1LU69 | Protein Rps27a-ps12 | 109,79 |
| M0RCU2 | Protein Rrbp1 | 72,86 |
| M0RBS6 | Protein Rrbp1 | 72,86 |
| B2RZD1 | Protein Sec61b | 180,22 |
| D3ZD11 | Protein Spcs2 | 274,89 |
| D3Z854 | Protein Tti1 | 81,17 |
| Q4QQV0 | Protein Tubb6 | 121,37 |
| D4A2P7 | Similar to Brain protein 44-like (Predicted) | 128,16 |
| P07632 | Superoxide dismutase [Cu-Zn] | 421,28 |
| P46462 | Transitional endoplasmic reticulum ATPase | 101,1 |
| Q5XIF6 | Tubulin alpha-4A chain | 105,57 |
| P62982 | Ubiquitin-40S ribosomal protein S27a | 109,79 |
| P62986 | Ubiquitin-60S ribosomal protein L40 | 109,79 |
| Q9Z1A6 | Vigilin | 115,02 |

The identified proteins unique in Control group are organized according to the alphabetical order. ^a^Identification is based on protein ID from UniProt protein database (<http://www.uniprot.org/>).

Supplementary Table 3. Proteins exclusively present in rats submandibular glands in Hg (MeHg) group.

| *^a^*Access number | Protein name description | PLGS Score |
| --- | --- | --- |
| P06761 | 78 kDa glucose-regulated protein | 416,41 |
| Q09073 | ADP/ATP translocase 2 | 315,62 |
| M0R8M9 | Heat shock cognate 71 kDa protein | 316,02 |
| P14659 | Heat shock-related 70 kDa protein 2 | 60,85 |
| Q63279 | Keratin, type I cytoskeletal 19 | 164,41 |
| Q64335 | Killer cell lectin-like receptor subfamily G member 1 | 189,51 |
| F1LTD7 | Protein Dennd4c | 421,22 |
| F1LM05 | Protein LOC299282 | 121,86 |
| D3ZFB2 | Protein Luc7l3 | 420,9 |
| Q6MG75 | Protein Nelfe | 253,66 |
| F1M5J3 | Protein Nipa1 | 222,49 |
| Q9EPJ1 | Protein Twist1 | 168,85 |
| P05544 | Serine protease inhibitor A3L | 121,86 |
| P68370 | Tubulin alpha-1A chain | 191,53 |
| Q6P9V9 | Tubulin alpha-1B chain | 170,38 |
| Q6AYZ1 | Tubulin alpha-1C chain | 191,53 |

The identified proteins unique in Hg group are organized according to the alphabetical order. ^a^Identification is based on protein ID from UniProt protein database (<http://www.uniprot.org/>).

Supplementary Table 4. Proteins exclusively present in rats submandibular glands in control group.

| *^a^*Access number | Protein name description | PLGS Score |
| --- | --- | --- |
| P35213 | 14-3-3 protein beta/alpha | 285,92 |
| P62260 | 14-3-3 protein epsilon | 268,52 |
| P68511 | 14-3-3 protein eta | 268,52 |
| P61983 | 14-3-3 protein gamma | 268,52 |
| P68255 | 14-3-3 protein theta | 268,52 |
| P35434 | ATP synthase subunit delta, mitochondrial | 210,14 |
| P62161 | Calmodulin | 588,62 |
| M0R8J8 | Carboxylic ester hydrolase | 147,62 |
| G3V936 | Citrate synthase | 265,57 |
| Q8VHF5 | Citrate synthase, mitochondrial | 265,57 |
| P09605 | Creatine kinase S-type, mitochondrial | 221,42 |
| Q6MGB6 | E3 ubiquitin-protein ligase RING1 | 158,98 |
| P07323 | Gamma-enolase | 131,56 |
| M0R660 | Glyceraldehyde-3-phosphate dehydrogenase | 171,14 |
| P0CG51 | Polyubiquitin-B | 203,24 |
| F1LML2 | Polyubiquitin-C | 203,24 |
| F1M446 | Protein AI314180 | 349,26 |
| P11598 | Protein disulfide-isomerase A3 | 352,92 |
| F1LRA1 | Protein ERGIC-53 | 214,36 |
| Q5EBB0 | Protein LOC298795 | 268,52 |
| D3ZBG6 | Protein Ptrhd1 | 351,89 |
| F1LU69 | Protein Rps27a-ps12 | 203,24 |
| G3V9A3 | Protein Sfn | 268,52 |
| P18897 | SMR2 protein | 474,84 |
| Q9JK00 | Sodium channel subunit beta-3 | 193,86 |
| P06686 | Sodium/potassium-transporting ATPase subunit alpha-2 | 148,81 |
| P51514 | Transcription factor 12 | 447,77 |
| Q6MFY8 | Tripartite motif-containing protein 40 | 184,95 |
| Q4QRB4 | Tubulin beta-3 chain | 192,64 |
| P62982 | Ubiquitin-40S ribosomal protein S27a | 203,24 |
| P62986 | Ubiquitin-60S ribosomal protein L40 | 203,24 |

The identified proteins unique in Control group are organized according to the alphabetical order. ^a^Identification is based on protein ID from UniProt protein database (<http://www.uniprot.org/>).

Supplementary Table 5. Proteins exclusively present in rats sublingual glands in Hg (MeHg) group.

| *^a^*Access number | Protein name description | PLGS Score |
| --- | --- | --- |
| P06761 | 78 kDa glucose-regulated protein | 433,49 |
| P62161 | Calmodulin | 770,24 |
| Q68FY0 | Cytochrome b-c1 complex subunit 1, mitochondrial | 122,57 |
| P63018 | Heat shock cognate 71 kDa protein | 674,38 |
| P14659 | Heat shock-related 70 kDa protein 2 | 541,83 |
| Q00729 | Histone H2B type 1-A | 239,59 |
| P01836 | Ig kappa chain C region, A allele | 434,5 |
| P13084 | Nucleophosmin | 321,07 |
| P10111 | Peptidyl-prolyl cis-trans isomerase A | 618,4 |
| Q63081 | Protein disulfide-isomerase A6 | 117,63 |
| Q3T1I4 | Protein PRRC1 | 243,35 |
| D3Z810 | Protein RGD1564129 | 230,52 |
| M0RCB1 | Uncharacterized protein | 618,84 |

The identified proteins unique in Control group are organized according to the alphabetical order. ^a^Identification is based on protein ID from UniProt protein database (<http://www.uniprot.org/>).

Supplementary Table 6. Proteins exclusively present in rats sublingual glands in control group.

| *^a^*Access number | Protein name description | PLGS Score |
| --- | --- | --- |
| M0RCB1 | Uncharacterized protein | 618,84 |
| Q4FZV3 | Axonemal dynein light intermediate polypeptide 1 | 160,18 |
| P02466 | Collagen alpha-2(I) chain | 250,32 |
| P00406 | Cytochrome c oxidase subunit 2 | 218,87 |
| P97541 | Heat shock protein beta-6 | 266,23 |
| P01835 | Ig kappa chain C region, B allele | 644,39 |
| Q5BJY9 | Keratin, type I cytoskeletal 18 | 162,58 |
| D3ZAP1 | Mediator of RNA polymerase II transcription, subunit 19 homolog (Yeast) (Predicted) | 291,11 |
| F1LQ08 | Protein Car6 | 621,25 |
| D3ZE71 | Protein Faap24 | 168 |
| F1LZM0 | Protein Foxn2 | 139,63 |
| F1M3U4 | Protein Gm15294 | 227,93 |
| M0R5K7 | Protein Pradc1 | 190,66 |
| D4A3I2 | Protein RGD1308117 | 145,8 |
| E9PSN4 | Protein Zc3h13 | 135,79 |
| P12346 | Serotransferrin | 235,78 |
| P06685 | Sodium/potassium-transporting ATPase subunit alpha-1 | 171,71 |
| Q07984 | Translocon-associated protein subunit delta | 271,56 |
| Q6QMY6 | Tsukushin | 123,23 |
| P68370 | Tubulin alpha-1A chain | 449,61 |
| Q6P9V9 | Tubulin alpha-1B chain | 449,61 |
| Q6AYZ1 | Tubulin alpha-1C chain | 449,61 |

The identified proteins unique in Control group are organized according to the alphabetical order. ^a^Identification is based on protein ID from UniProt protein database (<http://www.uniprot.org/>).
