# Supplementary material for: Oxidative Biochemistry Disbalance and Changes on Proteomic Profile in Salivary Glands of Rats Induced by Chronic Exposure to Methylmercury
Source: Oxid Med Cell Longev. 2017 Jul 24;2017:5653291. doi: 10.1155/2017/5653291 (PMC5546058; doi:10.1155/2017/5653291)
Supplement: Supplementary file 2 [file 5653291.f2.pdf]

Supplementary Table 1. Proteins exclusively present in rat parotid glands in MeHg (Hg) group.

[a]Access number	Protein name description	PLGS Score
P68511	14-3-3 protein eta	175,01
P61983	14-3-3 protein gamma	117,35
P63102	14-3-3 protein zeta/delta	146,33
Q08163	Adenylyl cyclase-associated protein 1	176,65
D3ZCV5	Aldehyde dehydrogenase, cytosolic 1	130,57
P62161	Calmodulin	401,4
G3V9M0	Cystatin	650,62
P06214	Delta-aminolevulinic acid dehydratase	130,7
Q641Y0	Dolichyl-diphosphooligosaccharide--protein glycosyltransferase 48 kDa subunit	271,08
M0RCH0	Eukaryotic translation initiation factor 3 subunit I	237,45
B0BNA7	Eukaryotic translation initiation factor 3 subunit I	237,45
P82995	Heat shock protein HSP 90-alpha	113,76
P34058	Heat shock protein HSP 90-beta	216,52
P56574	Isocitrate dehydrogenase [NADP], mitochondrial	131,56
P67779	Prohibitin	153,08
O88767	Protein deglycase DJ-1	414,81
Q5EBB0	Protein LOC298795	117,35
Q8K3F3	Protein phosphatase 1 regulatory subunit 14B	247,45
G3V834	Protein PRRC1	138,28
G3V9A3	Protein Sfn	117,35
P51647	Retinal dehydrogenase 1	133,52
Q66X93	Staphylococcal nuclease domain-containing protein 1	112,93
Q3MIE4	Synaptic vesicle membrane protein VAT-1 homolog	264,18
Q9JKC9	Synergin gamma	116,86
Q64428	Trifunctional enzyme subunit alpha, mitochondrial	145,78

The identified proteins unique in Hg group are organized according to the alphabetical order. [a]Identification is based on protein ID from UniProt protein database (http://www.uniprot.org/).

Supplementary Table 2. Proteins exclusively present in rat parotid glands control group

[a]Access number	Protein name description	PLGS Score
P29266	3-hydroxyisobutyrate dehydrogenase, mitochondrial	174,83
P13471	40S ribosomal protein S14	114,97
P38983	40S ribosomal protein SA	250,76
G3V880	Aa1017	121,63
G3V6S2	Aconitate hydratase	95,99
P00330	Alcohol dehydrogenase 1	3123,4
Q8VHE9	All-trans-retinol 13,14-reductase	156,89
P49088	Asparagine synthetase [glutamine-hydrolyzing]	87,56
Q499T7	Cilia- and flagella-associated protein 20	202,78
P02454	Collagen alpha-1(I) chain	93,85
Q62737	Cytochrome b-245 light chain	71,92
Q68FY0	Cytochrome b-c1 complex subunit 1, mitochondrial	105,14
Q63270	Cytoplasmic aconitate hydratase	91,63
Q68FU3	Electron transfer flavoprotein subunit beta	131,56
Q68FR9	Elongation factor 1-delta	132,39
Q99PF5	Far upstream element-binding protein 2	71,94
P04906	Glutathione S-transferase P	558,96
P62828	GTP-binding nuclear protein Ran	216,61
Q3KRF2	High density lipoprotein binding protein (Vigilin)	110,19
B4F777	High mobility group nucleosome-binding domain-containing protein 5	141,91
M0RCB8	Histone H3	155,2
Q6IFW6	Keratin, type I cytoskeletal 10	119,33
Q6IFV1	Keratin, type I cytoskeletal 14	125,11
Q6IFV3	Keratin, type I cytoskeletal 15	105,3
Q6IFU8	Keratin, type I cytoskeletal 17	122,8
Q6IFX1	Keratin, type I cytoskeletal 24	102,47
Q6IFU7	Keratin, type I cytoskeletal 42	105,3
Q6IG12	Keratin, type II cytoskeletal 7	116,62
O88989	Malate dehydrogenase, cytoplasmic	178,2
B1WC26	N-acetylneuraminic acid synthase	265,94
G3V8R1	Nucleobindin 2, isoform CRA_b	131,59
Q9JI85	Nucleobindin-2	131,59
Q5U317	Pre-mRNA 3'-end-processing factor FIP1	100,68

D4ADC7	Protein Adgra2	75,19
G3V6Y7	Protein Caap1	204,58
F1M5A1	Protein Calr4	127,07
Q63081	Protein disulfide-isomerase A6	142,66
D3ZN42	Protein Fam122b	169,96
F1MAF7	Protein Krt33a	159,6
Q6IFV6	Protein Krt35	123,6
Q6IFV5	Protein Krt36	102,47
M0RCJ8	Protein Krt78	79,76
F1MAC2	Protein Krt78	79,76
Q6PDV6	Protein LOC100911847	114,97
M0R6R9	Protein LOC102549011	281,46
D3ZL21	Protein LOC685619	93,55
Q6MG75	Protein Nelfe	112,12
F1LP37	Protein Nxnl1	75,75
B5DF04	Protein Rad51	116,75
F1LU69	Protein Rps27a-ps12	109,79
M0RCU2	Protein Rrbp1	72,86
M0RBS6	Protein Rrbp1	72,86
B2RZD1	Protein Sec61b	180,22
D3ZD11	Protein Spcs2	274,89
D3Z854	Protein Tti1	81,17
Q4QQV0	Protein Tubb6	121,37
D4A2P7	Similar to Brain protein 44-like (Predicted)	128,16
P07632	Superoxide dismutase [Cu-Zn]	421,28
P46462	Transitional endoplasmic reticulum ATPase	101,1
Q5XIF6	Tubulin alpha-4A chain	105,57
P62982	Ubiquitin-40S ribosomal protein S27a	109,79
P62986	Ubiquitin-60S ribosomal protein L40	109,79
Q9Z1A6	Vigilin	115,02

The identified proteins unique in Control group are organized according to the alphabetical order. [a]Identification is based on protein ID from UniProt protein database (http://www.uniprot.org/).

Supplementary Table 3. Proteins exclusively present in rats submandibular glands in Hg (MeHg) group.

[a]Access number	Protein name description	PLGS Score
P06761	78 kDa glucose-regulated protein	416,41
Q09073	ADP/ATP translocase 2	315,62

M0R8M9	Heat shock cognate 71 kDa protein	316,02
P14659	Heat shock-related 70 kDa protein 2	60,85
Q63279	Keratin, type I cytoskeletal 19	164,41
Q64335	Killer cell lectin-like receptor subfamily G member 1	189,51
F1LTD7	Protein Dennd4c	421,22
F1LM05	Protein LOC299282	121,86
D3ZFB2	Protein Luc7l3	420,9
Q6MG75	Protein Nelfe	253,66
F1M5J3	Protein Nipa1	222,49
Q9EPJ1	Protein Twist1	168,85
P05544	Serine protease inhibitor A3L	121,86
P68370	Tubulin alpha-1A chain	191,53
Q6P9V9	Tubulin alpha-1B chain	170,38
Q6AYZ1	Tubulin alpha-1C chain	191,53

The identified proteins unique in Hg group are organized according to the alphabetical order. [a]Identification is based on protein ID from UniProt protein database (http://www.uniprot.org/).

Supplementary Table 4. Proteins exclusively present in rats submandibular glands in control group.

[a]Access number	Protein name description	PLGS Score
P35213	14-3-3 protein beta/alpha	285,92
P62260	14-3-3 protein epsilon	268,52
P68511	14-3-3 protein eta	268,52
P61983	14-3-3 protein gamma	268,52
P68255	14-3-3 protein theta	268,52
P35434	ATP synthase subunit delta, mitochondrial	210,14
P62161	Calmodulin	588,62
M0R8J8	Carboxylic ester hydrolase	147,62
G3V936	Citrate synthase	265,57
Q8VHF5	Citrate synthase, mitochondrial	265,57
P09605	Creatine kinase S-type, mitochondrial	221,42
Q6MGB6	E3 ubiquitin-protein ligase RING1	158,98
P07323	Gamma-enolase	131,56
M0R660	Glyceraldehyde-3-phosphate dehydrogenase	171,14
P0CG51	Polyubiquitin-B	203,24
F1LML2	Polyubiquitin-C	203,24
F1M446	Protein AI314180	349,26
P11598	Protein disulfide-isomerase A3	352,92
F1LRA1	Protein ERGIC-53	214,36

Q5EBB0	Protein LOC298795	268,52
D3ZBG6	Protein Ptrhd1	351,89
F1LU69	Protein Rps27a-ps12	203,24
G3V9A3	Protein Sfn	268,52
P18897	SMR2 protein	474,84
Q9JK00	Sodium channel subunit beta-3	193,86
P06686	Sodium/potassium-transporting ATPase subunit alpha-2	148,81
P51514	Transcription factor 12	447,77
Q6MFY8	Tripartite motif-containing protein 40	184,95
Q4QRB4	Tubulin beta-3 chain	192,64
P62982	Ubiquitin-40S ribosomal protein S27a	203,24
P62986	Ubiquitin-60S ribosomal protein L40	203,24

The identified proteins unique in Control group are organized according to the alphabetical order. [a]Identification is based on protein ID from UniProt protein database (http://www.uniprot.org/).

Supplementary Table 5. Proteins exclusively present in rats sublingual glands in Hg (MeHg) group.

[a]**Access number**	**Protein name description**	**PLGS Score**
P06761	78 kDa glucose-regulated protein	433,49
P62161	Calmodulin	770,24
Q68FY0	Cytochrome b-c1 complex subunit 1, mitochondrial	122,57
P63018	Heat shock cognate 71 kDa protein	674,38
P14659	Heat shock-related 70 kDa protein 2	541,83
Q00729	Histone H2B type 1-A	239,59
P01836	Ig kappa chain C region, A allele	434,5
P13084	Nucleophosmin	321,07
P10111	Peptidyl-prolyl cis-trans isomerase A	618,4
Q63081	Protein disulfide-isomerase A6	117,63
Q3T1I4	Protein PRRC1	243,35
D3Z810	Protein RGD1564129	230,52
M0RCB1	Uncharacterized protein	618,84

The identified proteins unique in Control group are organized according to the alphabetical order. [a]Identification is based on protein ID from UniProt protein database (http://www.uniprot.org/).

Supplementary Table 6. Proteins exclusively present in rats sublingual glands in control group.

[a]Access number	Protein name description	PLGS Score
M0RCB1	Uncharacterized protein	618,84
Q4FZV3	Axonemal dynein light intermediate polypeptide 1	160,18
P02466	Collagen alpha-2(I) chain	250,32
P00406	Cytochrome c oxidase subunit 2	218,87
P97541	Heat shock protein beta-6	266,23
P01835	Ig kappa chain C region, B allele	644,39
Q5BJY9	Keratin, type I cytoskeletal 18	162,58
D3ZAP1	Mediator of RNA polymerase II transcription, subunit 19 homolog (Yeast) (Predicted)	291,11
F1LQ08	Protein Car6	621,25
D3ZE71	Protein Faap24	168
F1LZM0	Protein Foxn2	139,63
F1M3U4	Protein Gm15294	227,93
M0R5K7	Protein Pradc1	190,66
D4A3I2	Protein RGD1308117	145,8
E9PSN4	Protein Zc3h13	135,79
P12346	Serotransferrin	235,78
P06685	Sodium/potassium-transporting ATPase subunit alpha-1	171,71
Q07984	Translocon-associated protein subunit delta	271,56
Q6QMY6	Tsukushin	123,23
P68370	Tubulin alpha-1A chain	449,61
Q6P9V9	Tubulin alpha-1B chain	449,61
Q6AYZ1	Tubulin alpha-1C chain	449,61

The identified proteins unique in Control group are organized according to the alphabetical order. [a]Identification is based on protein ID from UniProt protein database (http://www.uniprot.org/).
